# Genotypic composition and performance of pea-nodulating rhizobia from soils outside the native plant-host range

**DOI:** 10.3389/fmicb.2023.1201140

**Published:** 2023-07-04

**Authors:** Junjie Zhang, Nan Wang, Shuo Li, Brigitte Brunel, Jingqi Wang, Yufeng Feng, Tao Yang, Xuxiao Zong

**Affiliations:** ^1^College of Food and Bioengineering, Zhengzhou University of Light Industry, Zhengzhou, Henan Province, China; ^2^Collaborative Innovation Center for Food Production and Safety of Henan Province, Zhengzhou, Henan Province, China; ^3^LSTM, Univ Montpellier, CIRAD, INRAE, Institut Agro Montpellier, IRD, Montpellier, France; ^4^Institute of Crop Sciences, Chinese Academy of Agricultural Sciences, Beijing, China

**Keywords:** *Pisum sativum*, *Rhizobium*, symbiosis, genetic diversity, *nodC gene*, symbiovar viciae

## Abstract

Cultivated soils need to shelter suitable rhizobia for legume cropping, especially in areas outside of the plant-host native range, where soils may lack efficient symbiotic partners. We analyzed the distribution patterns and traits of native rhizobia associated with *Pisum sativum* L. in soils of Hebei Province, a region that has recently experienced an expansion of pea production in China. A total of 43 rhizobial isolates were obtained from root-nodules and characterized genetically and symbiotically. The isolates discriminated into 12 genotypes as defined by PCR-RFLP of IGS DNA. Multiple locus sequence analysis (MLSA) based on the 16S rRNA, *recA*, *atpD* and *gyrB* of representative strains placed them into five clusters of four defined species (*R. sophorae*, *R. indicum*, *R. changzhiense*, and *R. anhuiense*) and a novel *Rhizobium* genospecies. *R. sophorae* was the dominant group (58%) followed by *R. indicum* (23%). The other groups composed of *R. changzhiense* (14%), *R. anhuiense* (1 isolate) and the new genospecies (1 isolate), were minor and site-specific. Based on *nodC* phylogeny, all representatives were intermingled within the symbiovar viciae with *R. sophorae* and *R. changzhiense* being a new record. All the tested strains showed efficient symbiotic fixation on pea plants, with half of them exhibiting better plant biomass performance. This suggests that the pea-nodulating rhizobia in Hebei Province form a specific community of efficient symbiotic rhizobia on pea, distinct from those reported in other countries.

## Introduction

Pea (*Pisum sativum* L.) is one of the most significant pulse grains around the world, accounting for 16% of global pulse production (Drew et al., 2012) due to the high protein content (up to around 30%) and quality of its seeds (Hacisalihoglu et al., 2020). The pea is believed to be native to the Middle and Near East, where domestication began about 10,000–9,000 years B.C. It then spread progressively to other countries with settlements (Smýkal et al., 2012). Chinese peas are thought to have originated from domestication in the Middle East, and introduced in China via an ancient trade road through southern Asia (Wu et al., 2017). As an exogenous species, the pea has been widely cultivated in China for over 2,000 years. Currently, it is grown on an area of about 2.4 million ha with a total production of 12.9 million t of both dry and fresh peas per year (FAOSTAT, 2022).

This legume species has been recorded as a nodule-forming plant since the 1880s in North European soils (Nutman, 1987) and its microsymbiont, *Rhizobium leguminosarum*, has been the earliest reported member of rhizobia studied since that time. Soil-dwelling rhizobia are capable of fixing dinitrogen in symbiosis with legumes by inducing undetermined nodules on pea roots (Oldroyd et al., 2011). This symbiotic relationship is based on a balance of nutrient exchanges between the partners. Plants supply carbohydrates (primarily succinate and malate, products of photosynthesis) and microhabitats to compatible bacteria, while bacteria differentiated into bacteroids supply ammonia [products of biological nitrogen fixation (BNF)] by secretion to plant hosts (Oldroyd et al., 2011; Rogel et al., 2011; Udvardi and Poole, 2013; Schulte et al., 2021).

The oldest described and most studied species *R. leguminosarum* was further classified in three symbiovars (sv.) depending on the host plants and symbiotic gene phylogeny: sv. viciae infects plants of tribe *Vicieae*, like pea and vetches (*Vicia* spp.); sv. trifolii forms nodules with clovers (*Trifolium* spp.) and sv. phaseoli, with common beans (*Phaseolus vulgaris* L; Rahi et al., 2020). Subsequently, pea-nodulating rhizobia were found in species *Rhizobium indicum* (Rahi et al., 2020), *Rhizobium ruizarguesonis* (Jorrin et al., 2020), *Rhizobium pisi* (Ramirez-Babena et al., 2008), *Rhizobium anhuiense* (Zhang et al., 2015), *Rhizobium laguerreae* (Flores-Félix et al., 2020), and *Ensifer meliloti* (Ilahi et al., 2021), all belonging to the sv. viciae. Except *E. meliloti, R. pisi*, *R. indicum* and *R. anhuiense,* all other species belonged to the *R. leguminosarum* complex (Rlc; Young et al., 2021), a group of closely related species that could only be differentiated by phylogeny of multilocus (or concatenated) sequence analysis (MLSA) of housekeeping genes, but not 16S rRNA genes (de Lajudie et al., 2021; Gurkanli, 2021). The presence or relative occurrence of these species varied according to different geographical aereas often at region or country scales depending on soil and crop history. For instance, *R. laguerreae* was found dominant in both northwest Spain (100% of the isolates) and Tunisia (gsR, 56%; Flores-Félix et al., 2020; Ilahi et al., 2021), while it formed a widespread minor group in Turkey (12.5%; Gurkanli, 2021). In Turkey, the most common pea microsymbiont was the *R. leguminosarum* complex genospecies B (Rlc gsB, 47.5%) followed by *R. ruizarguesonis* (Rlc gsC, 20%). In addition, genospecies Rlc gsA and Rlc gsE were found as site-specific groups nodulating pea in Turkey (Gurkanli, 2021) and were not recovered in Tunisia (Ilahi et al., 2021). These previous studies demonstrated that diversity and uneven distribution of rhizobial communities associated with pea plants showed distinct geographic patterns, shaped by the adaptation of rhizobia to local conditions that may impact symbiosis interaction and functioning (Wang et al., 2019).

Application of chemical fertilizers has effectively enhanced crop yields, but their long-term excessive use has led to increased costs, reduced fertilizer efficiency in agricultural production, as well as severe environmental and biodiversity degradation (Zhang et al., 2012; Olivares et al., 2013). Consequently, the employment of rhizobial strains as inoculants to replace commonly used N-fertilizers has become of primary importance and a goal to be achieved for economic and environmental implications (Mia and Shamsuddin, 2010; Bhardwaj et al., 2014). In recent years, the exploitation and improvement of BNF of field pea is a key element for eco-sustainable agriculture (Holt-Gimenez and Altieri, 2013) based upon its worldwide cultivation and high BNF efficiency. It was estimated that more than half of the biologically fixed nitrogen worldwide is yielded by *Rhizobium-legume* symbioses (Coyne, 2005; Erana, 2021). BNF in pea crops can reach one of the highest levels with more than 80% of the N provided to plants, while an average of only 25–35 kg/ha of N is introduced in the soil, depending on the tillage and cropping system. Therefore, isolation and phenotypic screening of rhizobial strains to select as bio-inoculants is a crucial strategy. Such screening is also important because the efficiency of the symbiosis can be affected by many other factors, like host-specificity and the ability of the selected strain to compete with local rhizobia (Abi-Ghanem et al., 2013; Bourion et al., 2018). The plant growth benefits provided to the same legume species by different rhizobial strains at a given location can vary up to 10-fold (Denison and Kiers, 2004).

Considering all the aforementioned aspects, and the fact that pea-nodulating rhizobia in China have not been systematically studied since the extensive revision of the *Rhizobium* and Rlc taxonomic system, we decided to conduct the present study. The aim of this work was to evaluate the diversity, relative abundance, and geographic distribution of native rhizobia that nodulate *P. sativum* in Hebei Province, a non-native area of pea. Traditionally, the pea has been grown sparingly in Hebei as an economic crop for fresh vegetables and grains, and the pea cultivation area has been rapidly expanded there to meet consumer demand. For this purpose, the taxonomic status of the isolated strains from root-nodules was determined through ribosomal intergenic typing, phylogenies analyses of housekeeping genes (*recA*, *atpD*, and *gyrB*), the 16S rRNA gene and a symbiotic gene (*nodC*). Additionally, the distribution of rhizobia in relation to soil properties and the potential of representative strains to induce effective symbiosis, were also investigated.

## Materials and methods

### Field and soil sampling and soil characterization

Soils were sampled from three fields cultivated with local pea in the Guyuan County, Hebei Province of China. These sites were at the Guyuan Institute of Crop Science, Chinese Academy of Agricultural Sciences (E 115°39′, N 41°40′12″, altitude 1,410 m); Xiaochang town (E 115°46′48″, N 41°24′36″, altitude 1,519 m); and Dashila village (E 115°54′, N41°30′36″, altitude 1,415 m). At each site, soils were sampled to a depth of 10–20 cm from the fields during the flowering stage of *Pisum sativum* in June 2020. For each site, five randomly taken soil sub-samples of equal volume were thoroughly mixed to constitute a representative soil sample of the field site. Then, soil samples were crushed to a uniform state, and transported to the laboratory in an ice-filled cooler (Zhang et al., 2018). Part of each representative soil sample was chemically analyzed for pH, total salts (TS), organic matter (OM), available phosphorus (AP), available potassium (AK) and total nitrogen (TN) as described previously (Appunu et al., 2009; Han et al., 2009).

### Rhizobial isolation and conservation

Surface-sterilized seeds (2.5% w/v NaClO solution for 5 min) of pea (variety of Zhongqin No.1) were germinated and the seeding was sown in surface-sterilized plastic pots (15 cm high × 10 cm diameter) filled with a soil-sterilized vermiculite mixture (1/5 vol/vol) as described previously (Zhang et al., 2019). All plants were grown under greenhouse conditions of 25/20°C (day/night) with a 16-h photoperiod. Sterilized water was added to the pots throughout the experiment as required. After 45 days, all plants in all soils were up-rooted and bacterial strains were isolated from nodules according to the standard protocol (Zhang et al., 2019). Five plants from each soil treatment were selected for the collection of root nodules and isolation of rhizobia. Root nodules were surface sterilized and the individual sterilized root nodule was crushed in sterile water and the bacterial suspension was streaked onto a Yeast extract-Mannitol Agar (YMA) plate. After incubation at 28°C for 3 to 5 days, single colonies representing the dominant bacteria in each plate were picked up and purified by cross-streaking on new YMA plates until pure cultures were obtained. All purified isolates were conserved in tryptone yeast (TY) broth (tryptone 5 g; yeast extract 3 g; CaCl_2_ 0.6 g; distilled water 1 L, pH 7.0) supplied with glycerol (20%, v/v) at −80°C for long-term storage and on YMA slants at 4°C for temporary storage.

### Genomic fingerprinting of Rhizobial isolates

Genomic DNA (gDNA) of each rhizobial isolate was extracted according to Terefework et al. (2001). The gDNA was estimated qualitatively and quantitatively by using a nanodrop (Thermo Fisher) and high quality gDNA was used as template for PCR amplifications of the 16S-23S rRNA intergenic spacer (IGS) region with primers IGS1490’ (forward) and IGS132’ (reverse; Laguerre et al., 1996). PCR amplification was carried out in a standard 50 μL reaction mixture including 1 μL of DNA template and 5 U of *Taq* DNA polymerase (Sangon Biotech (Shanghai) Co., Ltd.). Aliquots of amplified PCR products were visualized after electrophoresis in a 1.0% (w/v) agarose gel labeled with GoldView type I. Then PCR products were digested separately with the endonucleases *HaeIII*, *MspI* and *HhaI* (Laguerre et al., 1994) at 37°C for 10 h. The 16S-23S rDNA IGS type of each strain was designated after separation and visualization of restriction fragments by electrophoresis in 2.5% (w/v) agarose gel and UV-illumination.

### Molecular and phylogenetic identification of the isolates

**Figure 1:**
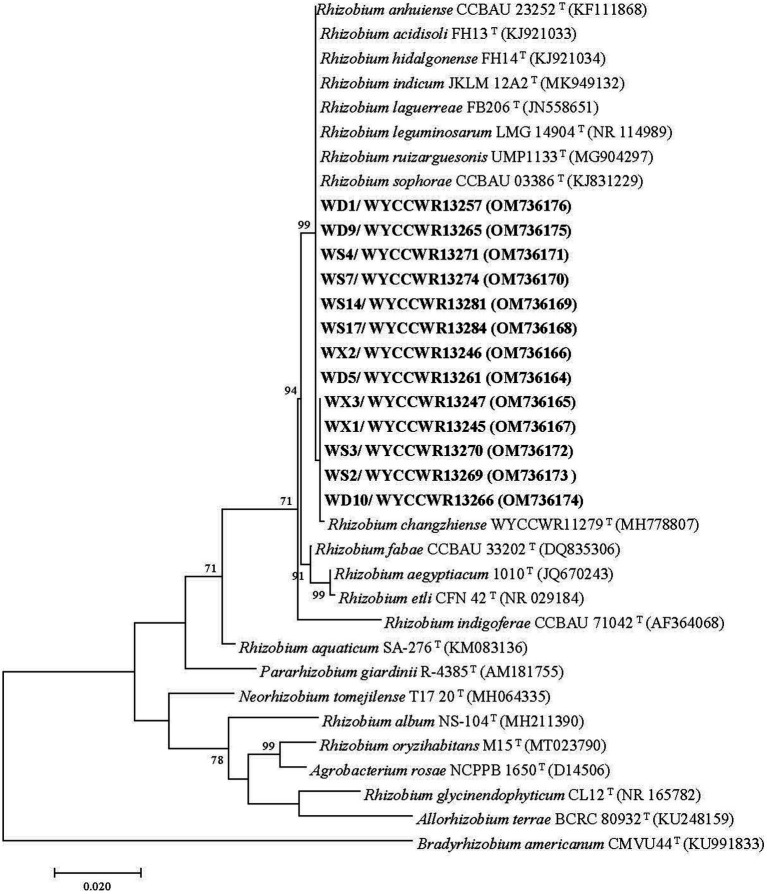
Maximum-likelihood phylogenetic tree based on 16S rRNA gene sequences (1,217 base pairs) from studied *Pisum sativum* L. rhizobia and related rhizobial sequences retrieved from Genbank. The tree was inferred under the best-fit model (T92 + G + I) and rooted with *Bradyrhizobium americanum* CMVU44^T^. Scale bar indicates 0.02 nucleotide substitutions per site. Bootstrap confidence values (%) calculated for 500 replications >70% are shown at the internodes.

**Figure 2:**
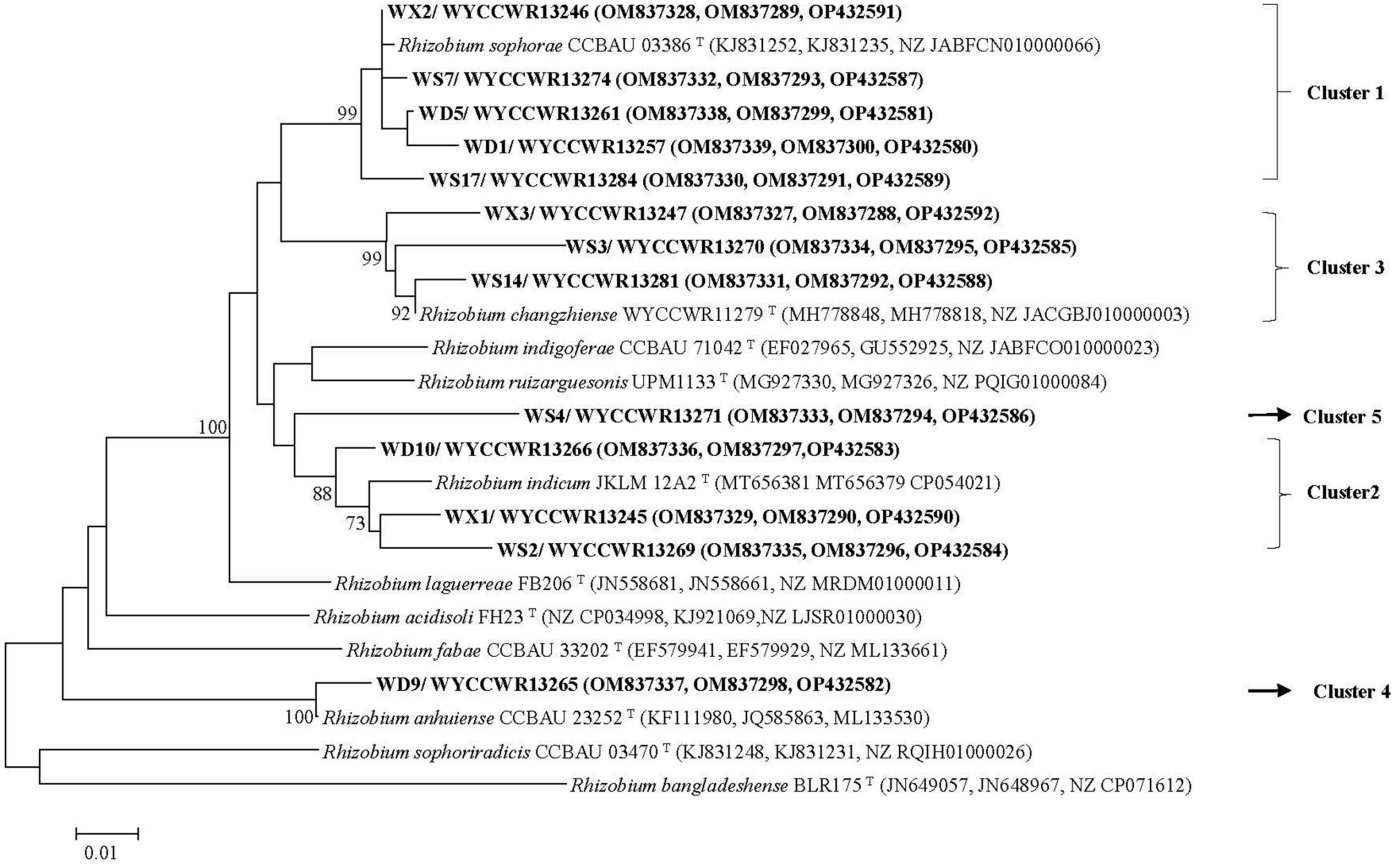
Maximum-likelihood phylogenetic tree based on concatenated *recA-atpD-gyrB* gene sequences (1,023 base pairs) showing the relationships of rhizobia isolated from studied *Pisum sativum* L. in Hebei Province of China. The tree was constructed under the best-fit model (T92 + G). Scale bar indicates 0.010 nucleotide substitution per site. Bootstrap confidence values (%) calculated for 500 replications >70% are indicated at the internodes.

Isolates sharing the same RFLP pattern of 16S-23S rDNA IGS in this study were designed as an IGS type. One representative strain for each IGS type in each sample site was selected for amplification of the 16S rRNA gene using the forward primer P1 and reverse primer P6 (Laguerre et al., 1996). The PCR products were verified as mentioned above, and were sent for commercial sequencing (Sangon Biotech (Shanghai) Co., Ltd.). The acquired sequences were compared in the NCBI database using the online BLASTN tool and the sequences for type strains of defined *Rhizobium* species sharing similarities greater than 97.0% with the new isolates were extracted. The phylogenetic analysis was conducted in the MEGA 7.0 software (Kumar et al., 2016). Sequences were aligned using Clustal W and the best model of sequence evolution was selected. Then the phylogenetic tree was inferred by using the Maximum-likelihood (ML) and the non-parametric bootstrap methods (500 pseudo-replications). DNA fragments of *recA* (coding for DNA recombination protein), *atpD* (encoding for ATP synthase beta chain) and *gyrB* (structural gene for DNA gyrase beta subunit) were amplified separately by PCR using the primer pairs recA41F/recA640R, atpD255F/atpD782R and AMgyrBf/AMgyrBr, respectively, (Vinuesa et al., 2005; Stepkowski et al., 2012). The nodulation gene *nodC* was amplified by using the primer pair nodC540F/nodC1160R (Sarita et al., 2005). The PCR conditions were adopted from previous reports (Sarita et al., 2005). PCR products verification, sequencing and tree construction of each sequenced gene were performed as mentioned above. Furthermore, sequences of *atpD, recA* and *gyrB* genes were concatenated and aligned using Clustal W (Thompson et al., 1997). Distance calculation, construction of the concatenated housekeeping gene ML tree, bootstrap analysis were performed in MEGA 7.0 as described above. The sequences resulted from the alignment of the related genes have been deposited in the NCBI database (accession numbers are indicated on the trees, Figures 1, 2; Supplementary Figures S1–S5).

### Correlation analysis between soil properties and Rhizobial communities

A distance-based redundancy analysis (RDA) was performed using CANOCO software version 4.54 to investigate the relationships between soil properties (TN, AP, AK, OM, TS, and pH) and the rhizobial community composition based on IGS genotypes.

### Nodulation, cross-inoculation and symbiotic efficiency test

The representative strains of the IGS types were tested for their ability to nodulate their original host plant *P. sativum* and also *Vicia faba* L., *Vicia sativa* L., *Vigna radiata* L., *Glycine max* L., *Arachis hypogaea* L. and *Cicer arietinum* L. Symbiotic efficiency of the strains was tested on pea. Briefly, surface sterilized seedlings were aseptically transferred in pots (1 plant/pot) containing sterile vermiculite as substrate and inoculated with 1 mL of rhizobial suspension (OD_600_ = 1.0). Plants were grown under greenhouse conditions and watered with sterile N-free nutrient solution as required (Zhang et al., 2012). Nodulation was evaluated 40 days after inoculation for all plant species above. Pea growth was estimating by weighting their dry root and shoot biomass and measuring leaf chlorophyll contents (SPAD chlorophyll meter). Uninoculated plants as a negative control, were included and all plant treatments were performed in three replicates. Data were analyzed by one-way ANOVA followed by an LSD *post hoc* test (*p* = 0.001).

## Results

### Physicochemical characteristics of soils

All three sites differed from each other in their levels of organic matter, total nitrogen, available phosphorus, and available potassium (Table 1). Field soil at H-DSL contained the highest contents of OM (41.4 g/kg soil), TN (2.5 g/kg soil) and AK (195 mg/kg soil) while soil from site H-ES contained the highest AP (57.8 mg / kg soil). At the opposite, site H-XC exhibited the lowest proportions of organic carbon and nitrogen, and available P and K minerals in soil. Two of the sites had weakly alkaline soil pH values of 7.5, while the remaining site had a neutral pH of 7.1. All three sites had comparable and very low total soluble (TS) contents of 0.6–0.7 g/kg of soil.

**Table 1:** Soil properties from the different sites studied.

Sampling site	Soil trait					
	OM (g/kg)	TN (g/kg)	AP (mg/kg)	AK (mg/kg)	pH_water_	TS (g/kg)
H-XC	26.4 ± 2.67 c	1.52 ± 0.13 c	8.6 ± 0.153 c	116 ± 4.60 c	7.5 ± 0.058 a	0.7 ± 0.116 a
H-DSL	41.4 ± 0.68 a	2.5 ± 0.085 a	51.4 ± 4.114 b	195 ± 6.11 a	7.1 ± 0 b	0.7 ± 0.116 a
H-ES	30.3 ± 1.36 b	1.91 ± 0.125 b	57.8 ± 1.308 a	150 ± 6.11 b	7.5 ± 0.058 a	0.6 ± 0.100 a

OM is organic matter; TN is total nitrogen; AP is available phosphate; AK is available potassium; TS is total salts.

H-XC refers to Xiaochang town, Hebei Province; H-DSL refers to Dashila village, Hebei Province; H-ES refers to Experimental site, Hebei Province.

Data in the table are the mean value and its standard deviation (three replicates). Different letters in a column indicated that the difference between different soil properties reached a 0.05 significant level (*p* ≤ 0.05).

### IGS PCR-RFLP analysis

Totally, 43 rhizobial isolates were obtained from in 3 locations. The 43 isolates were distinguished into 6, 8, and 7 RFLP patterns obtained from the restriction enzymes *HaeIII*, *MspI* and *HhaI,* respectively. By combining all RFLP patterns, the IGS PCR-RFLP fingerprinting allowed the classification of all isolates into 12 IGS types (Table 2). Among the 12 IGS types, IGS type 1 represented the most abundant population (with 20 isolates), followed by IGS type 2 (5 isolates), type 3 (4 isolates), types 4 and 5 (3 isolates each), and type 6 (2 isolates). All the other IGS types have just 1 isolate. Isolates of IGS type 1 distributed over all the three sampling sites, with the H-ES site having the largest number (15 isolates, 34.9%) and the H-CX the smallest (1 isolate, 2.3%). IGS type 2 isolates were detected at sites H-DSL (3 isolates, 7.0%) and H-XC (2 isolates, 4.7%; Table 2). IGS types 3, 5, and 7 were only distributed in site H-XC (8 isolates, 18.6%); IGS types 4 and 8 were only found in H-DSL (4 isolates, 9.3%) and IGS types 6, 9, 10, 11, and 12 were only distributed in H-ES (6 isolates, 14.0%). Site H-ES was inhabited by the most diverse *P. sativum*-nodulating rhizobial community with 6 IGS types, while sites H-XC and H-DSL harbored 5 and 4 IGS types, respectively, (Table 2), indicating that the richness and evenness of rhizobial IGS types varied across the sampling area.

**Table 2:** Genetic groupings of *Rhizobium* isolates associated with *Pisum sativum* and their geographical distribution in the different sampling sites.

Representative isolate (WYCCWR no.)	MLSA similarity^b^ with (%)					Isolates/IGS type	Distribution [number of strains (%)] of IGS types in the sampling site			Subtotal
	Rso	Rin	Rch	Ran	Rru		H-XC^c^	H-DSL	H-ES	
*Rhizobium sophorae* (Cluster 1, C1)^a^										58.1%, 25 isolates, 3 IGS types, 3 sites
13,246 (WX2)	99.8	96.9	96.4	92.4	96.2	1/7	1 (2.3)	0	0	
13,261 (WD5)	99.3	97.0	96.3	92.8	96.5	5/1	1 (2.3)	4 (9.3)	0	
13,257 (WD1)	98.6	96.3	95.5	92.1	95.7	3/4	0	3 (7.0)	0	
13,274 (WS7)	99.4	96.7	96.2	92.3	96.0	1/11	0	0	1 (2.3)	
13,284 (WS17)	98.5	96.4	96.7	92.0	95.7	15/1	0	0	15 (34.9)	
*Rhizobium indicum* (Cluster 2, C2)										23.4%, 10 isolates, 3 IGS types, 3 sites
13,266 (WD10)	96.7	97.9	96.1	92.6	96.8	5/2	2 (4.7)	3 (7.0)	0	
13,245 (WX1)	96.3	98.1	95.3	92.2	96.2	3/5	3 (7.0)	0	0	
13,269 (WS2)	95.5	97.3	94.7	91.3	95.5	2/6	0	0	2 (4.7)	
*Rhizobium changzhiense* (Cluster 3, C3)										13.9%, 6 isolates, 3 IGS types, 2 sites
13,247 (WX3)	95.2	94.4	98.1	91.4	94.4	4/3	4 (9.3)	0	0	
13,270 (WS3)	94.1	93.6	97.1	90.5	93.4	1/9	0	0	1 (2.3)	
13,281 (WS14)	95.7	94.8	99.2	91.6	95.1	1/12	0	0	1 (2.3)	
*Rhizobium anhuiense* (Cluster 4, C4)										2.3%
13,265 (WD9)	91.5	92.2	91.6	99.1	91.6	1/8	0	1 (2.3)	0	
*Rhizobium* sp. I (Cluster 5, C5)										2.3%
13,271 (WS4)	94.9	94.8	94.4	91.5	94.7	1/10	0	0	1 (2.3)	
Total number of isolates/IGS types and strains (%) per site						43/12	11 (25.6)	11 (25.6)	21 (48.8)	100%
Proportion of genomic species in all the isolates: clusters (%)							C1 (4.6) C2 (11.7) C3 (9.3)	C1 (16.3) C2 (7.0) C4 (2.3)	C1 (37.2) C2 (4.7) C3 (4.7) C5 (2.3)	

^a^ Clusters or genomic species were defined by MLSA (recA – atpD – gyrB).

^b^ MLSA similarity with the most related strains: Rso, R. sophorae CCBAU 03386^T^; Rin, R. indicum JKLM 12A2^T^; Rch, R. changzhiense WYCCWR 11279^T^; Ran, R. anhuiense CCBAU 23252^T^; Rru, R. ruizarguesonis UPM1133^T^.

^c^ H-XC, Xiaochang town, Hebei province; H-DSL, Dashila village, Hebei province; H-ES, Experimental site, Hebei province.

### Phylogenetic analysis of core and symbiotic genes

Nearly full-length 16S rRNA genes were successfully amplified and sequenced for 13 rhizobial isolates representing all 12 IGS types and sites of origin (Table 2). All representative isolates clustered together with several defined *Rhizobium* species in a single clade that showed 99.8–100% similarity in their 16S rRNA gene sequences. This clade comprises the type strains of *R. hidalgonense* FH14^T^, *R. anhuiense* CCBAU 23252^T^, *R. indicum* JKLM 12A2^T^, *R. laguerreae* FB206^T^, *R. leguminosarum* LMG 14904^T^, *R. ruizarguesonis* UMP1133^T^, *R. sophorae* CCBAU 03386^T^, *Rhizobium acidisoli* FH13 ^T^ and *R. changzhiense* WYCCWR 11279^T^ (Figure 1).

The representative isolates divided into five clades (C1–C5) in the phylogenetic tree based on their concatenated *recA-atpD-gyrB* sequences (Figure 2). Five representative isolates from IGS types 1, 4, 7, and 11 grouped together in C1 cluster which also included the type strain of *R. sophorae*. They shared 98.5–99.8% similarities with each other and similarities ≤97.0% with all type strains of other closely related *Rhizobium* species (Table 2). Thus, cluster C1 was identified as *R. sophorae.* Cluster C2 contained *R. indicum* JKLM 12A2^T^ and IGS types 2, 5, and 6 representing 10 isolates (23% in total) which shared 97.3–98.1% similarities with each other and less than 96.7% similarities with the other *Rhizobium* type strains; they were therefore identified as *R. indicum*. Cluster C3 (6 isolates from 3 IGS types) showed 97.1–99.2% similarities with each other and with *R. changzhiense* WYCCWR 11279^T^, while these isolates presented less than 95.7% similarities with the other *Rhizobium* species; so, this cluster was identified as *R. changzhiense*. Clusters C4 and C5 contained only the IGS type 10 and IGS type 8 respectively, each covering a single isolate. The C4 isolate WYCCWR 13265 was identified as belonging to the species *R. anhuiense* since it displayed 99.1% similarity with type strain CCBAU 23252^T^. Finally, the C5 isolate WYCCWR 13271 had similarities of less than 94.9% with all type strains of the closest defined *Rhizobium* species (Table 2; Supplementary Figure S4) and was therefore assigned to a novel *Rhizobium* genospecies. The phylogenetic analyses of the single gene of *recA, atpD*, and *gyrB* were shown in Supplementary Figures S1–S3.

**Figure 3:**
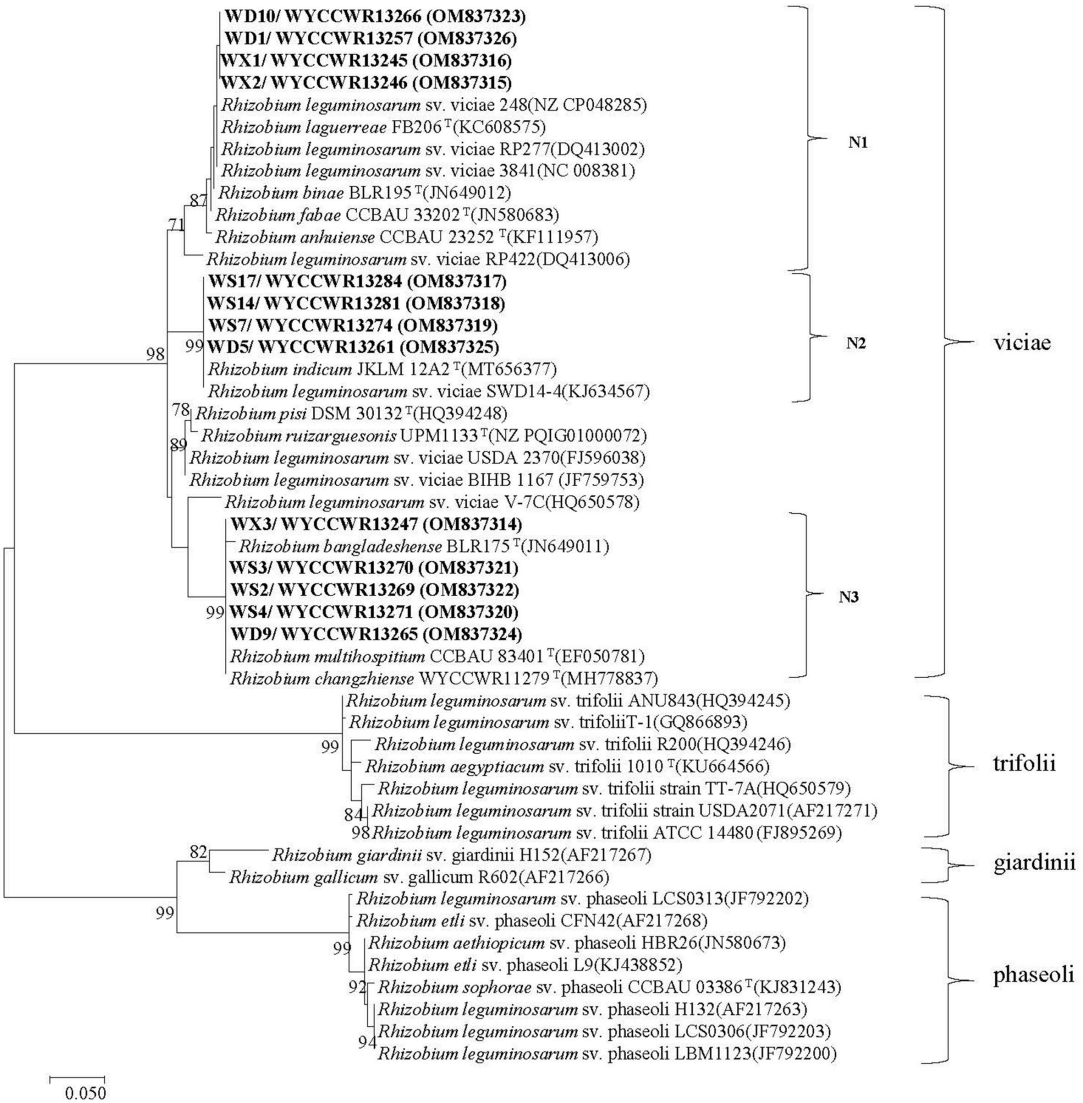
Maximum-likelihood phylogenetic tree based on symbiotic gene nodC (355 base pairs) showing the relationships of the rhizobia isolated from nodules of *Pisum sativum* L. in Hebei Province of China. The tree was constructed using the maximum-likelihood method under the best-fit model (T92 + I). Scale bar indicates 0.05 nucleotide substitutions per site. Bootstrap confidence values (%) calculated for 500 replications >70% are indicated at the internodes.

The *nodC* genes were amplified from all the 13 representative isolates, confirming their genetic basis for rhizobial symbiosis. As shown in the *nodC* phylogeny, three strongly supported groups (N1–N3) were defined among the representatives (Figure 3). First, symbiotype N1 clustered together with *nodC* sequences from strains *R. binae* BLR195^T^, *R. fabae* CCBAU 33202^T^, *R. anhuiense* CCBAU 23252^T^, *R. laguerreae* FB206^T^, *Rlv* 3,841, and *Rlv* 248 with similarity values higher than 98.3%. Second, symbiotype N2 contained *nodC* sequences 100% similar to those of *R. indicum* JKLM12A2^T^ and *Rlv* sv. viciae SWD14-4. Finally, symbiotype N3 encompassed *nodC* sequences 100% similar to those of *R. multihospitium* CCBAU 83401^T^ or *R. changzhiense* WYCCWR 11279^T^, and 99.1% similar to the *nodC* of *R. bangladeshense* BLR175^T^ (Figure 3).

The *nodC* and concatenated *recA-atpD-gyrB* gene trees were not congruent. Symbiotype N1 covered four representatives belonging to C1 (*R. sophorae*) and C2 (*R. indicum*) with 100% *nodC* phylogeny similarities. Representative isolates of symbiotype N2 that displayed 100% *nodC* phylogeny similarities, belonged to C1 or C3. The single representative of C4 or C5 both belonged to the symbiotype N3 while other N3 strains were also part of C2 and C3 (Figure 2; Supplementary Figure S3; Table 2). It is worth noting that the three *nodC* types showed a variable distribution among the sampling sites: all representative strains isolated from H-DSL clustered in symbiotypes N1 to N3, representatives from H-XC in symbiotypes N1 and N3, and representatives from H-ES in symbiotypes N2 and N3. In this study, representatives of *R. changzhiense* (C3) were found in two *nodC* gene haplotypes (N2 and N3), while those of *R. sophorea* (C1) fell into *nodC* haplotypes N1 and N2, those of *R. indicum* (C2) into *nodC* haplotypes N1 and N3, and those of *R. anhuinense* (C4) and *Rhizobium* sp. I (C5) into *nodC* haplotype N3.

### Nodulation range and symbiotic efficiency of Rhizobial strains

All representatives from the 12 different IGS types nodulated *Pisum sativum, Vicia faba* and *Vicia sativa*. None of them nodulated *Vigna radiata*, *Glycine max*, *Arachis hypogaea* or *Cicer arietinum*. Compared to the uninoculated control (*p* < 0.001), representative isolates of the IGS types increased leaf chlorophyll units (+14–23%) and total plant dry weight (+45–86%) of pea indicating that they were effective rhizobial symbionts. Six strains (WYCCWR13245, −57, −61, 65, −70, −74) tended to show higher plant dry biomass (+72–86%), while four of them (WYCCWR13247, −69, −81, −84) exhibited significantly lower pea dry biomass (+45–53%; Figure 3; Table 3).

**Table 3:** Dry weight of the pea shoot by inoculation of each *Rhizobium* isolate.

Isolates	Average of Dry weight/g
Negative control	0.163 ± 0.007
WD1/WYCCWR13257	0.298 ± 0.012
WD5/WYCCWR13261	0.281 ± 0.003
WD9/WYCCWR13265	0.278 ± 0.010
WD10/ WYCCWR13266	0.264 ± 0.017
WS2/WYCCWR13269	0.240 ± 0.012
WS3/WYCCWR13270	0.230 ± 0.018
WS4/WYCCWR13271	0.269 ± 0.004
WS7/WYCCWR13274	0.293 ± 0.002
WS14/WYCCWR13281	0.241 ± 0.010
WS17/WYCCWR13284	0.246 ± 0.005
WX1/WYCCWR13245	0.283 ± 0.017
WX2/WYCCWR13246	0.268 ± 0.023
WX3/WYCCWR13247	0.236 ± 0.003

### Correlation analysis between Rhizobial distribution and soil properties

**Figure 4:**
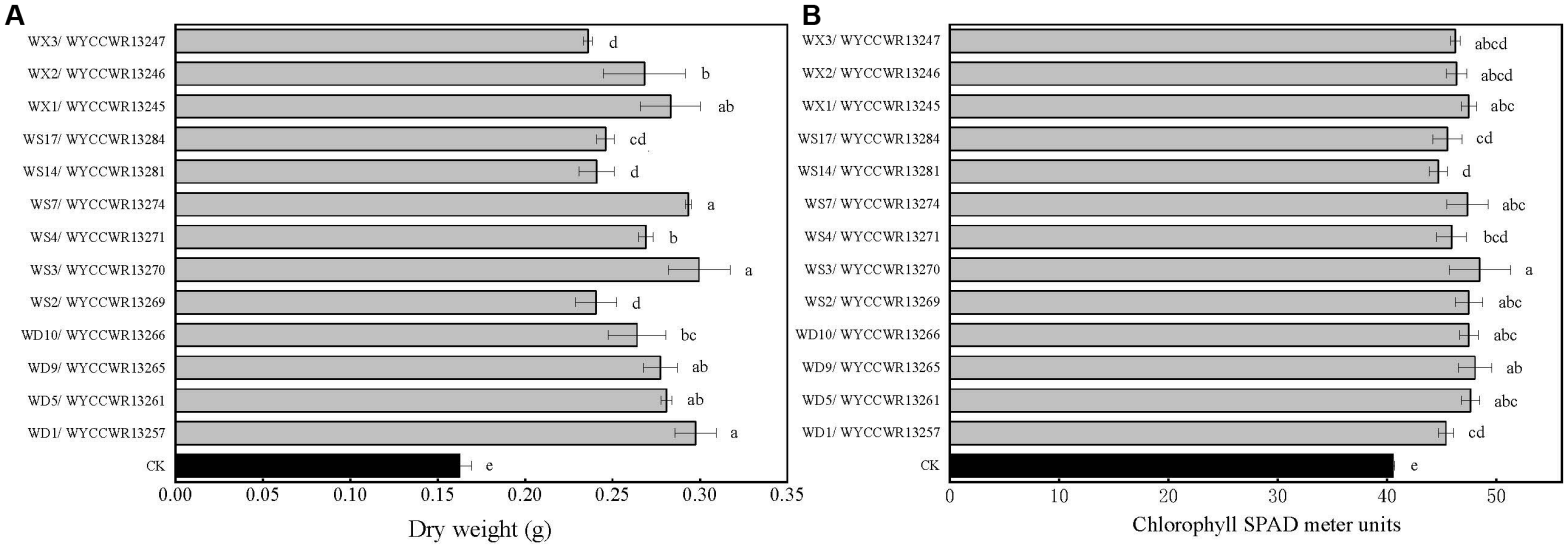
Symbiotic performance of 13 representative strains evaluated on pea grown under greenhouse conditions 40 days after inoculation, CK is the negative control (uninoculated plants). The experiment was conducted in triplicates. In addition, three subsamples were performed for each SPAD replication. **(A)** Dry shoot and root weight (g per plant). **(B)** Chlorophyll SPAD meter units. Different letters above bars show significant differences (ANOVA + LSD test; ***, *p* < 0.001). Bars indicate the standard error.

Redundancy analysis was used to explore the relationships between soil chemical properties and the rhizobial community composition based on IGS genotypes. The analysis was limited to 3 field sites with a narrow pH range (pH 7.1–7.5) and similar total salts across the sites. Nevertheless, the sites differed from each other, in particular, organic matter, total nitrogen, available phosphorus and available potassium were lower at Xiaochang town than that at Dashila village and the experimental site of the Guyuan Institute of Crop Science. According to RDA results (Figure 4), all ribosomal IGS types were separated into five distinct groups (Groups I to V) and soil chemical factors had different effects on the distribution of these rhizobial groups and IGS types. Group I includes the IGS types 4 and 8 encompassing 4 isolates. Its distribution was positively correlated with higher OM, AK, and TN values (Figure 4) that coincided with the soil properties of H-DSL (Table 2). Thus, nutriments with higher OM, TN and AK content (Table 1) appeared more suitable for the survival or *P. sativum* nodulation of Group I rhizobia. Group II (including IGS type 1) gathered the majority of isolates (20) distributed across the three sites (Table 2): it presented a positive association with low TS contents, higher AP, but intermediate OM, AK, and TN values (Figure 4). Group III was formed by five IGS types (6, 9, 10, 11 and 12) in a balanced population size (6 isolates) and found only in H-ES (Table 2). Meanwhile, strains belonging to Group III were correlated with higher AP and pH, but lower TN, AK, OM and TS (Figure 4). Group IV comprised rhizobia characterized by IGS types 3, 5 and 7, and trapped exclusively from site H-XC (Table 2): it was associated with higher pH and TS contents but lower OM, AK, TN, and AP values (Figure 4). Finally, isolates of Group V (including only IGS type 2 of sites H-XC and H-DSL) were positively distributed with higher TS, but lower AP and pH values. Thus, we found that the different rhizobial populations were influenced differently by edaphic factors, with a potential positive or negative correlation. According to the IGS type biogeography obtained in this study, the distribution of most strains belonging to *R. sophorae* (20/25 were in Group II) might be more suitable to higher AP, TN, AK and OM, while rhizobia of *R. changzhiense* (Group III and IV) could be favored by higher levels of soil pH. Besides, no obvious preference was shown for *R. indicum* (inside Groups III, IV, and V; Figure 4; Tables 1, 2). Thus, rhizobial species seemed to have different responses to soil chemical characteristics. Furthermore, the distribution pattern found at the scale of IGS genotypes may have occurred in the soil independently of that of the rhizobial species.

## Discussion

Although pea plants have been cultured widely in different ecoregions in China, the diversity and geographic distribution of pea-nodulating rhizobia in China have rarely been studied (Ramirez-Babena et al., 2008). Previously, *R. leguminosarum,* two unnamed Rlc genomic species and *R. etli* were detected using the phylogeny of 16S rRNA genes and PCR-amplified ribosomal IGS restriction patterns (Yang et al., 2008). More recently, *R. anhuiense* was described based on a polyphasic study (Ramirez-Babena et al., 2008) of pea-nodulating rhizobia in two subtropical regions of China (South East). Unlike the previous studies, we systematically investigated the diversity of *P. sativum* nodulating microsymbionts across three sampling sites within a temperate region of Hebei Province (North East China). Taking all the results of the 16S rRNA sequence analysis, the phylogeny of concatenated *recA-atpD-gyrB* gene sequences, IGS PCR-RFLP analysis, and the *nodC* gene phylogeny, all the isolates obtained in this study were classified as a diverse community consisting of 12 IGS types within 5 genomic species of the genus *Rhizobium,* all of which belong to the sv. viciae. The dominant species of pea-nodulating rhizobia in the three sampling sites were *R. sophorae* (58% in relative abundance) and *R. indicum* (23%), which were found to be distributed universally across all three sites. In contrast, *R. changzhiense*, *R. anhuiense*, and a novel *Rhizobium* genospecies within the Rlc were identified as minor groups and were site-specific. Based on the concatenated housekeeping gene tree, most of the isolates including the new *R. changzhiense* ones, belong to the complex Rlc (Olivares et al., 2013; de Lajudie et al., 2021; Young et al., 2021; Zhang et al., 2021) with only two isolates falling outside of it (*R. anhuiense* and the new genospecies C5). This provides new information on the diversity and geographic distribution of pea-nodulating rhizobia. Firstly, a unique community composition of pea-nodulating rhizobia was found in the temperate area studied in China, compared with previous reports in the subtropical zone in China (Ramirez-Babena et al., 2008) and other countries (Bergey et al., 1984; Ramirez-Babena et al., 2008; Jorrin et al., 2020; Rahi et al., 2020; Gurkanli, 2021; Ilahi et al., 2021; Young et al., 2021). Secondly, the identification of *R. sophorae* and *R. changzhiense* as novel pea-nodulating rhizobia expands the range of known rhizobial species associated with this plant. *R. sophorae* was initially isolated from effective nodules of the shrubby Sophora (*Sophora flavescens*) in Changzhi City (Shanxi Province, China), and was found to be able to effectively nodulated not only Sophora but also *Phaseolus vulgaris* (Jiao et al., 2015). Later, this rhizobial species was also recovered from root nodules of *Vicia faba* L. grown in Panxi (southwest China; Chen et al., 2018), southwest China (Zhang et al., 2019), and Hebei Province (northeast China) (Zhang et al., 2022). Our results extend the promiscuous nitrogen-fixing host plant list for *R. sophora* to include *P. sativum.* Together with the above-mentioned studies, these findings confirm the widespread existence of sv. viciae in *R. sophorae*. This emphasizes the wide distribution of this rhizobial species over space and time, as well as its high adaptability to soil and environmental factors in China. *R. changzhiense* was first isolated from *V. sativa* cultivated in the Changzhi city of China (Zhang et al., 2021). Together with our results, it provides evidence that rhizobial strains originated from both *Vicia sativa* (Zhang et al., 2021) and *P. sativum* belonged to the same species and symbiovar. *R. indicum* was isolated from root nodules of pea (*P. sativum*) cultivated in the Indian trans-Himalayas (Rogel et al., 2011). Meanwhile *R. anhuiense* was first found in rhizobia of pea and *V. faba* in Anhui and Jiangxi Provinces (Li et al., 2016), and has also been widely recorded in Shandong Province (Li et al., 2016). Our results in the present study might demonstrate that these species have a wide geographic distribution in the world and that pea plants have selected the chromosomal background or communities adapted to the local conditions during the long-term legume cultivation. Thirdly, the detection of highly conserved *nodC* genes across the 5 genomic species identified in this study offered further evidence that pea plants have stringently selected symbiosis genes in their microsymbionts. This selective pressure may favor symbiosis genes toward the most adapted indigenous rhizobia, as reported in other cases (He et al., 2011). Fourthly, our results also imply the necessity to screen out high-quality rhizobial strains with strong adaptability to local conditions to produce inoculants to increase the yield of pea or other legumes.

**Figure 5:**
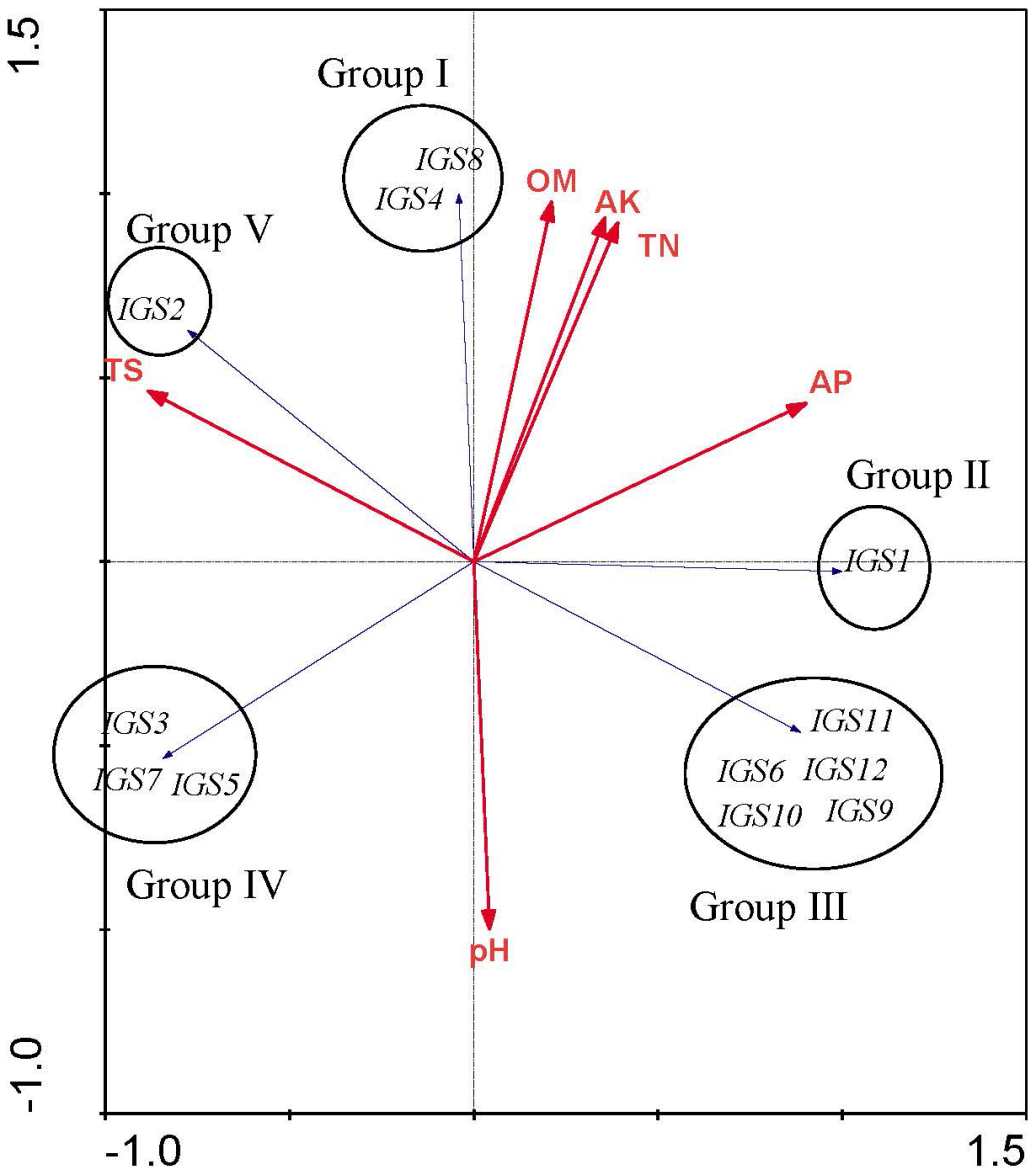
Redundancy analysis (RDA) to relate the distribution of the 12 IGS types of isolates to physicochemical factors of soils collected from the different sites. Blue arrows indicate IGS types of rhizobia and red arrows represent soil properties. The longer the arrow was, the greater the influence of the soil property presents on the distribution of the IGS types. The smaller the angle between the arrow and the IGS type was, the stronger the effect of the soil property on distribution of the IGS type.

The levels of OM, TN, AP, and AK among the three field soils sampled were significantly different in this study, and the distribution of IGS types was related to these soil characteristics. We also found that the same species isolated from different soil types had different responses to soil physicochemical characteristics, as evidenced by a distinct pattern observed at the IGS genotypes within species (Figure 5). Soil chemical factors had varying effects on the distribution of the rhizobial species and IGS types. For instance, we observed a positive correlation between IGS type 1 (*R. sophorae*) and a negative correlation of IGS types 3 (*R. changzhiense*), 5 (*R. indicum*), and 7 (*R. sophorae*) with AP, which may influence their separation into Groups II and IV (Figure 5). Similarly, a positive correlation of IGS type 2 (*R. indicum*) and a negative correlation of IGS types 6 (*R. indicum*), 9 and 12 (*R. changzhiense*), 10 (*Rhizobium* sp. I), and 11 (*R. sophorae*) with TS influenced their separation in two distribution patterns. These relationships were consistent with previous reports that soil traits were selective abiotic factors for the biogeography of rhizobial species or IGS types (Han et al., 2009).

In conclusion, our study demonstrated the existence of indigenous rhizobia that form an effective symbiosis with pea cultivated in North China, in which *R. sophorae* and *R. changzhiense* were new records as pea-nodulating rhizobia. *R. sophorae* was highly abundant in the studied soils (58%) and formed a unique species assemblage of pea-nodulating rhizobia along with four less frequent species or genospecies (*R. indicum*, *R. changzhiense, R. anhuiense* and a novel genospecies). Moreover *R. sophorae* and *R. indicum* occurred in all tested soil types, indicating that strains of these species could be better competitors and adapt to different soil conditions. Finally, all strains belonged to the sv. viciae, regardless of their species affiliation. The genetic diversity of pea-associated rhizobia from Hebei Province provided a significant guideline for local pea cultivation and breeding efforts, as well as for the site-specific selection of efficient rhizobial genetic types.

## Data availability statement

The datasets presented in this study can be found in online repositories. The names of the repository/repositories and accession number(s) can be found in the article/Supplementary material.

## Author contributions

JZ and XZ designed the experiment. JZ wrote the manuscript. NW and SL done most of the experiment. YF and JW done some of the experiment. TY and XZ took the soil samples from the farmland. BB and TY improved the manuscript. All authors contributed to the article and approved the submitted version.

## Funding

This work was financed by the National Key R&D Program of China (2021YFD1600602-02/YFD1600602) from XZ, Science and Technology Innovation Talents in Universities of Henan Province (22HASTIT035) from JZ and project of Sabbatical Year SIP20200726 authorized by IPN, Mexico from ET Wang.

## Conflict of interest

The authors declare that the research was conducted in the absence of any commercial or financial relationships that could be construed as a potential conflict of interest.

## Publisher’s note

All claims expressed in this article are solely those of the authors and do not necessarily represent those of their affiliated organizations, or those of the publisher, the editors and the reviewers. Any product that may be evaluated in this article, or claim that may be made by its manufacturer, is not guaranteed or endorsed by the publisher.
