# Supplementary material for: Genotypic composition and performance of pea-nodulating rhizobia from soils outside the native plant-host range
Source: Front Microbiol. 2023 Jul 4;14:1201140. doi: 10.3389/fmicb.2023.1201140 (PMC10353855; doi:10.3389/fmicb.2023.1201140)
Supplement: Supplementary file 1 [file Presentation_1.PPTX]

## Slide 1
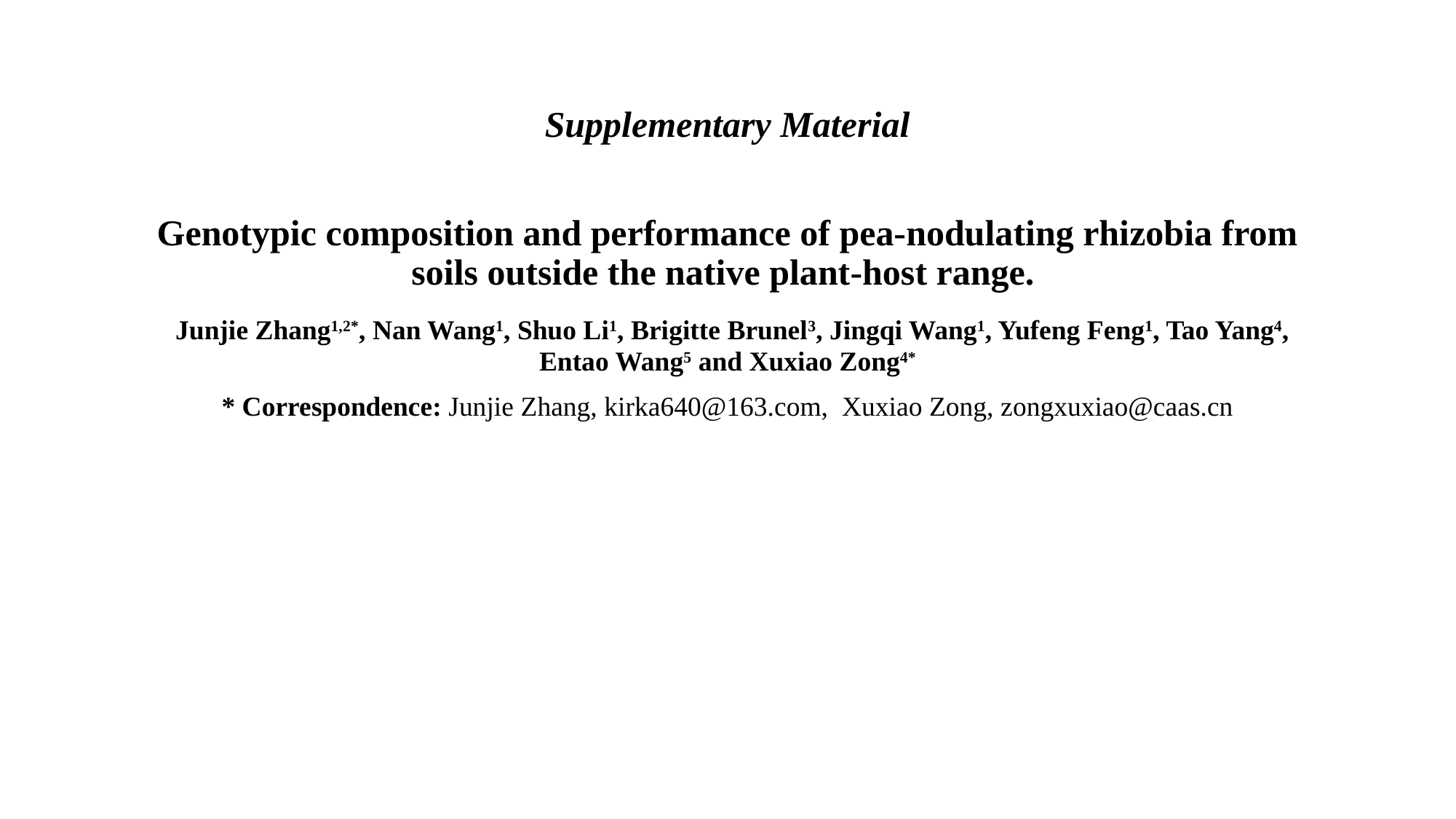

Supplementary Material
Genotypic composition and performance of pea-nodulating rhizobia from soils outside the native plant-host range.
 Junjie Zhang1,2*, Nan Wang1, Shuo Li1, Brigitte Brunel3, Jingqi Wang1, Yufeng Feng1, Tao Yang4, Entao Wang5 and Xuxiao Zong4*
* Correspondence: Junjie Zhang, kirka640@163.com, Xuxiao Zong, zongxuxiao@caas.cn

## Slide 2
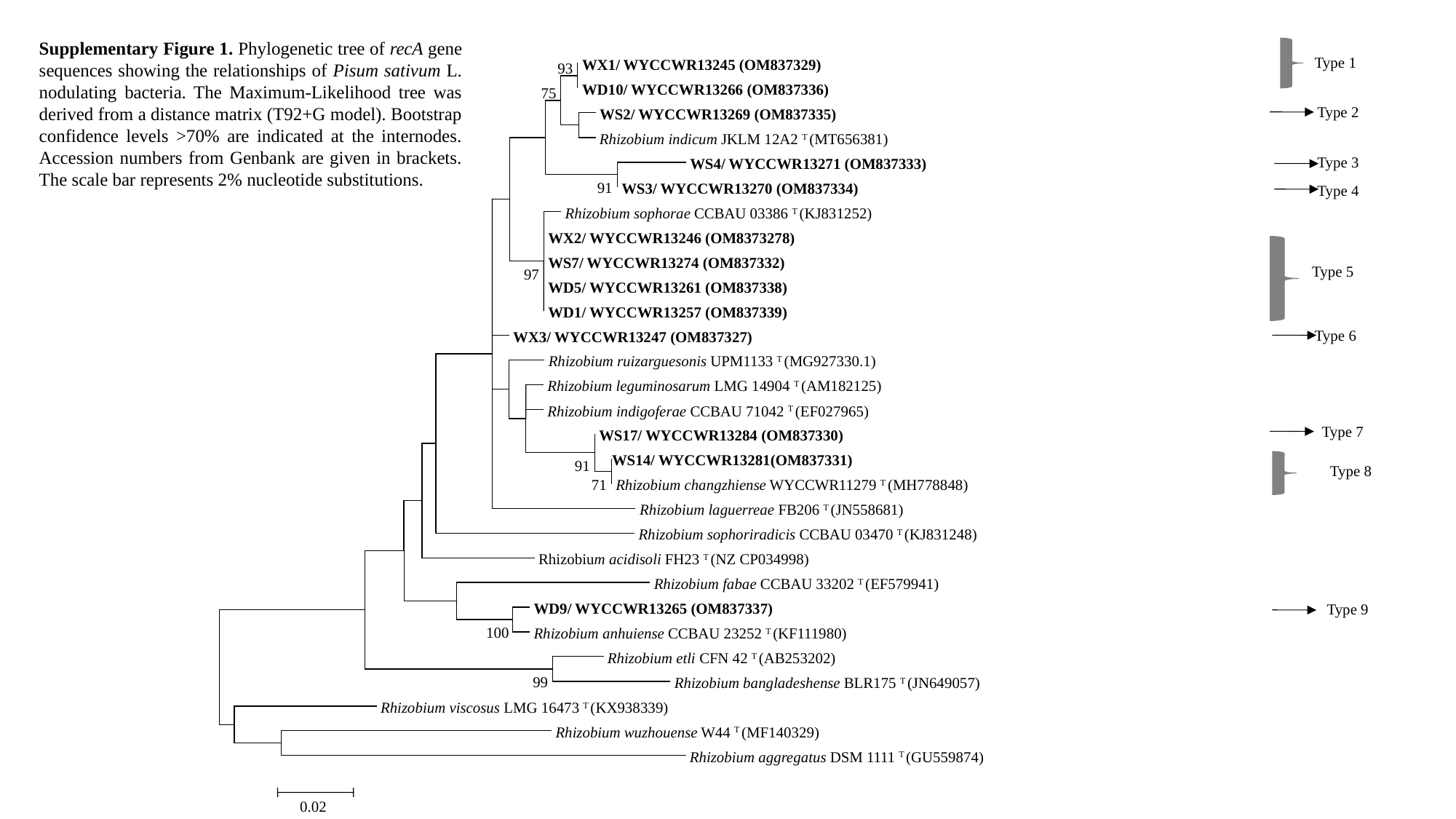

Supplementary Figure 1. Phylogenetic tree of recA gene sequences showing the relationships of Pisum sativum L. nodulating bacteria. The Maximum-Likelihood tree was derived from a distance matrix (T92+G model). Bootstrap confidence levels >70% are indicated at the internodes. Accession numbers from Genbank are given in brackets. The scale bar represents 2% nucleotide substitutions.
Type 1
 WX1/ WYCCWR13245 (OM837329)
93
 WD10/ WYCCWR13266 (OM837336)
75
Type 2
 WS2/ WYCCWR13269 (OM837335)
 Rhizobium indicum JKLM 12A2 T (MT656381)
Type 3
 WS4/ WYCCWR13271 (OM837333)
Type 4
91
 WS3/ WYCCWR13270 (OM837334)
 Rhizobium sophorae CCBAU 03386 T (KJ831252)
 WX2/ WYCCWR13246 (OM8373278)
 WS7/ WYCCWR13274 (OM837332)
Type 5
97
 WD5/ WYCCWR13261 (OM837338)
 WD1/ WYCCWR13257 (OM837339)
Type 6
 WX3/ WYCCWR13247 (OM837327)
 Rhizobium ruizarguesonis UPM1133 T (MG927330.1)
 Rhizobium leguminosarum LMG 14904 T (AM182125)
 Rhizobium indigoferae CCBAU 71042 T (EF027965)
Type 7
 WS17/ WYCCWR13284 (OM837330)
WS14/ WYCCWR13281(OM837331)
91
Type 8
71
 Rhizobium changzhiense WYCCWR11279 T (MH778848)
 Rhizobium laguerreae FB206 T (JN558681)
 Rhizobium sophoriradicis CCBAU 03470 T (KJ831248)
 Rhizobium acidisoli FH23 T (NZ CP034998)
 Rhizobium fabae CCBAU 33202 T (EF579941)
Type 9
 WD9/ WYCCWR13265 (OM837337)
100
 Rhizobium anhuiense CCBAU 23252 T (KF111980)
 Rhizobium etli CFN 42 T (AB253202)
99
 Rhizobium bangladeshense BLR175 T (JN649057)
 Rhizobium viscosus LMG 16473 T (KX938339)
 Rhizobium wuzhouense W44 T (MF140329)
 Rhizobium aggregatus DSM 1111 T (GU559874)
0.02

## Slide 3
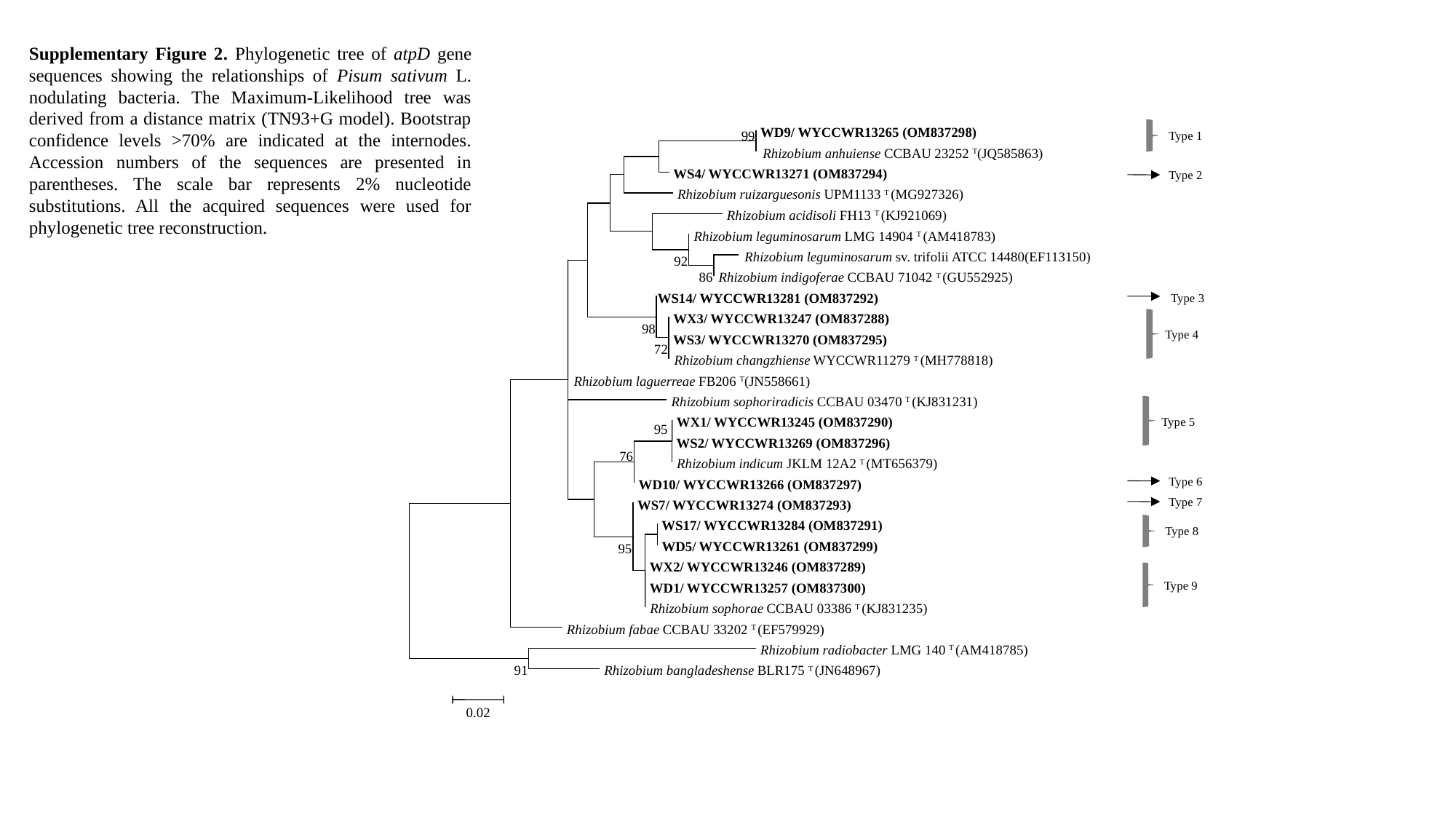

Supplementary Figure 2. Phylogenetic tree of atpD gene sequences showing the relationships of Pisum sativum L. nodulating bacteria. The Maximum-Likelihood tree was derived from a distance matrix (TN93+G model). Bootstrap confidence levels >70% are indicated at the internodes. Accession numbers of the sequences are presented in parentheses. The scale bar represents 2% nucleotide substitutions. All the acquired sequences were used for phylogenetic tree reconstruction.
Type 1
 WD9/ WYCCWR13265 (OM837298)
99
 Rhizobium anhuiense CCBAU 23252 T(JQ585863)
Type 2
 WS4/ WYCCWR13271 (OM837294)
 Rhizobium ruizarguesonis UPM1133 T (MG927326)
 Rhizobium acidisoli FH13 T (KJ921069)
 Rhizobium leguminosarum LMG 14904 T (AM418783)
 Rhizobium leguminosarum sv. trifolii ATCC 14480(EF113150)
92
86
 Rhizobium indigoferae CCBAU 71042 T (GU552925)
Type 3
WS14/ WYCCWR13281 (OM837292)
 WX3/ WYCCWR13247 (OM837288)
98
Type 4
 WS3/ WYCCWR13270 (OM837295)
72
 Rhizobium changzhiense WYCCWR11279 T (MH778818)
 Rhizobium laguerreae FB206 T(JN558661)
 Rhizobium sophoriradicis CCBAU 03470 T (KJ831231)
Type 5
 WX1/ WYCCWR13245 (OM837290)
95
 WS2/ WYCCWR13269 (OM837296)
76
 Rhizobium indicum JKLM 12A2 T (MT656379)
Type 6
 WD10/ WYCCWR13266 (OM837297)
Type 7
 WS7/ WYCCWR13274 (OM837293)
 WS17/ WYCCWR13284 (OM837291)
Type 8
 WD5/ WYCCWR13261 (OM837299)
95
 WX2/ WYCCWR13246 (OM837289)
Type 9
 WD1/ WYCCWR13257 (OM837300)
 Rhizobium sophorae CCBAU 03386 T (KJ831235)
 Rhizobium fabae CCBAU 33202 T (EF579929)
 Rhizobium radiobacter LMG 140 T (AM418785)
91
 Rhizobium bangladeshense BLR175 T (JN648967)
0.02

## Slide 4
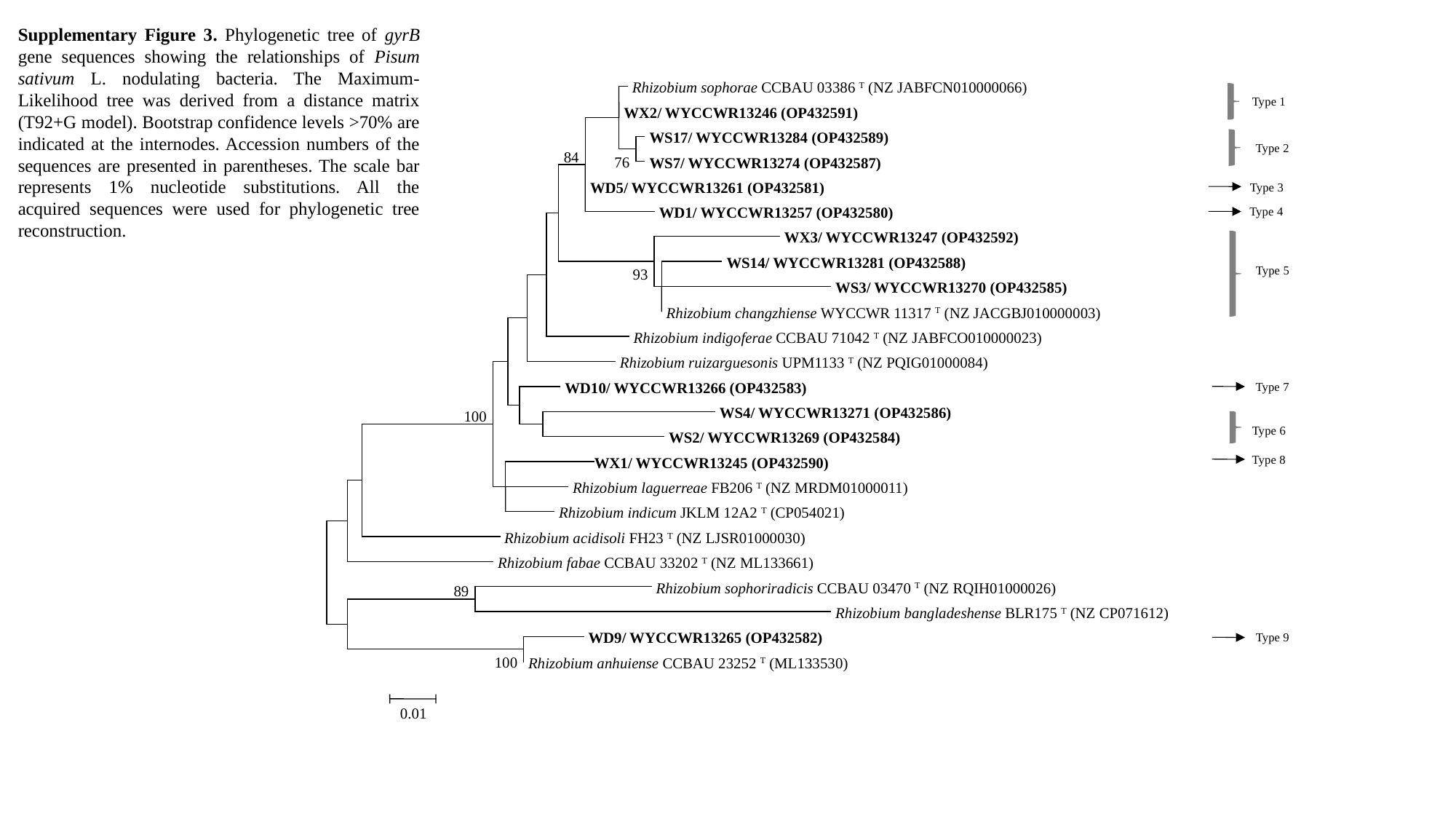

Supplementary Figure 3. Phylogenetic tree of gyrB gene sequences showing the relationships of Pisum sativum L. nodulating bacteria. The Maximum-Likelihood tree was derived from a distance matrix (T92+G model). Bootstrap confidence levels >70% are indicated at the internodes. Accession numbers of the sequences are presented in parentheses. The scale bar represents 1% nucleotide substitutions. All the acquired sequences were used for phylogenetic tree reconstruction.
 Rhizobium sophorae CCBAU 03386 T (NZ JABFCN010000066)
 WX2/ WYCCWR13246 (OP432591)
 WS17/ WYCCWR13284 (OP432589)
84
76
 WS7/ WYCCWR13274 (OP432587)
 WD5/ WYCCWR13261 (OP432581)
 WD1/ WYCCWR13257 (OP432580)
 WX3/ WYCCWR13247 (OP432592)
 WS14/ WYCCWR13281 (OP432588)
93
 WS3/ WYCCWR13270 (OP432585)
 Rhizobium changzhiense WYCCWR 11317 T (NZ JACGBJ010000003)
 Rhizobium indigoferae CCBAU 71042 T (NZ JABFCO010000023)
 Rhizobium ruizarguesonis UPM1133 T (NZ PQIG01000084)
 WD10/ WYCCWR13266 (OP432583)
 WS4/ WYCCWR13271 (OP432586)
100
 WS2/ WYCCWR13269 (OP432584)
WX1/ WYCCWR13245 (OP432590)
 Rhizobium laguerreae FB206 T (NZ MRDM01000011)
 Rhizobium indicum JKLM 12A2 T (CP054021)
 Rhizobium acidisoli FH23 T (NZ LJSR01000030)
 Rhizobium fabae CCBAU 33202 T (NZ ML133661)
 Rhizobium sophoriradicis CCBAU 03470 T (NZ RQIH01000026)
89
 Rhizobium bangladeshense BLR175 T (NZ CP071612)
 WD9/ WYCCWR13265 (OP432582)
100
 Rhizobium anhuiense CCBAU 23252 T (ML133530)
0.01
Type 1
Type 2
Type 3
Type 4
Type 5
Type 7
Type 6
Type 8
Type 9

## Slide 5
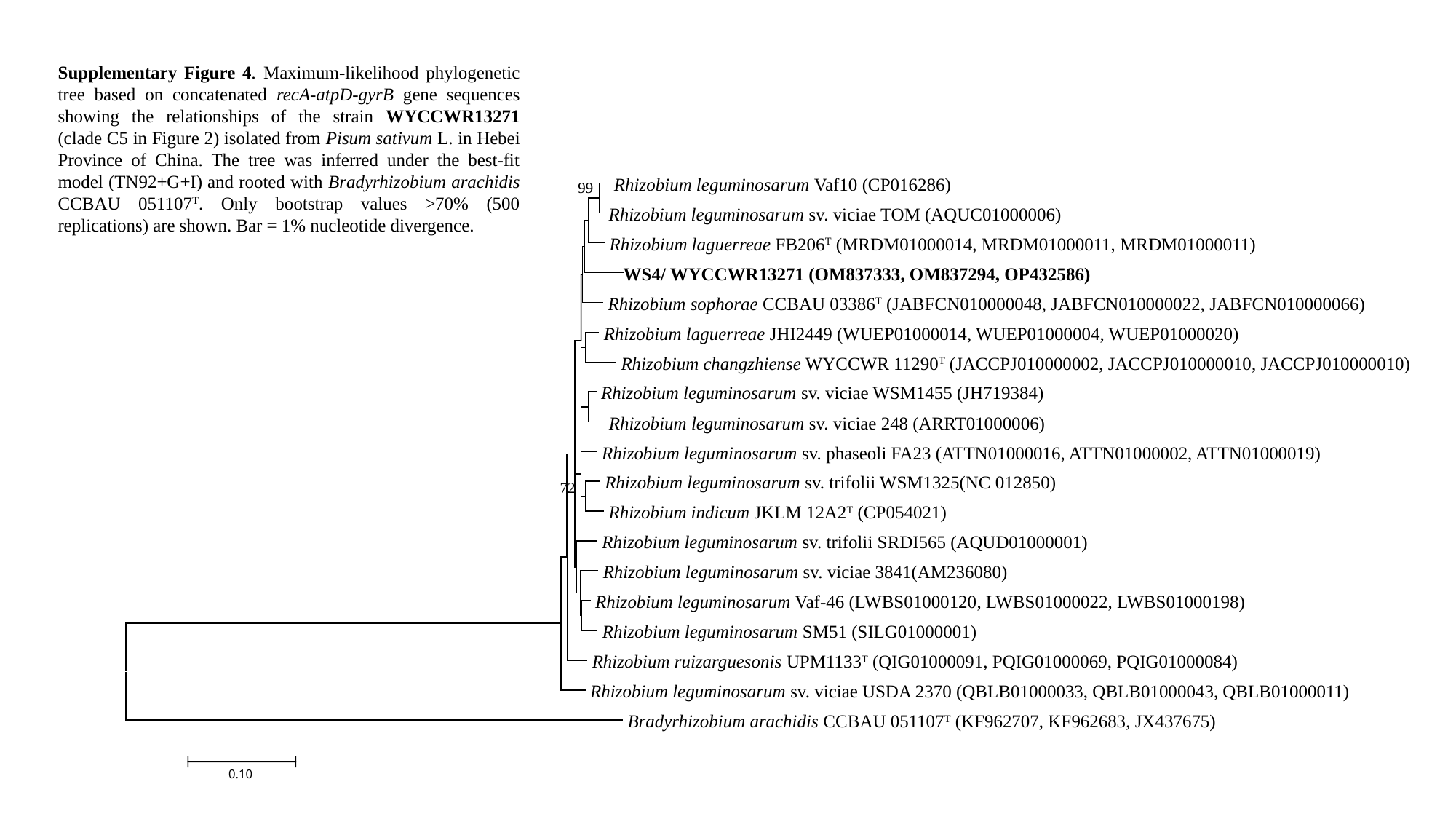

Supplementary Figure 4. Maximum-likelihood phylogenetic tree based on concatenated recA-atpD-gyrB gene sequences showing the relationships of the strain WYCCWR13271 (clade C5 in Figure 2) isolated from Pisum sativum L. in Hebei Province of China. The tree was inferred under the best-fit model (TN92+G+I) and rooted with Bradyrhizobium arachidis CCBAU 051107T. Only bootstrap values >70% (500 replications) are shown. Bar = 1% nucleotide divergence.
 Rhizobium leguminosarum Vaf10 (CP016286)
99
 Rhizobium leguminosarum sv. viciae TOM (AQUC01000006)
 Rhizobium laguerreae FB206T (MRDM01000014, MRDM01000011, MRDM01000011)
WS4/ WYCCWR13271 (OM837333, OM837294, OP432586)
 Rhizobium sophorae CCBAU 03386T (JABFCN010000048, JABFCN010000022, JABFCN010000066)
 Rhizobium laguerreae JHI2449 (WUEP01000014, WUEP01000004, WUEP01000020)
 Rhizobium changzhiense WYCCWR 11290T (JACCPJ010000002, JACCPJ010000010, JACCPJ010000010)
 Rhizobium leguminosarum sv. viciae WSM1455 (JH719384)
 Rhizobium leguminosarum sv. viciae 248 (ARRT01000006)
 Rhizobium leguminosarum sv. phaseoli FA23 (ATTN01000016, ATTN01000002, ATTN01000019)
 Rhizobium leguminosarum sv. trifolii WSM1325(NC 012850)
72
 Rhizobium indicum JKLM 12A2T (CP054021)
 Rhizobium leguminosarum sv. trifolii SRDI565 (AQUD01000001)
 Rhizobium leguminosarum sv. viciae 3841(AM236080)
 Rhizobium leguminosarum Vaf-46 (LWBS01000120, LWBS01000022, LWBS01000198)
 Rhizobium leguminosarum SM51 (SILG01000001)
 Rhizobium ruizarguesonis UPM1133T (QIG01000091, PQIG01000069, PQIG01000084)
 Rhizobium leguminosarum sv. viciae USDA 2370 (QBLB01000033, QBLB01000043, QBLB01000011)
 Bradyrhizobium arachidis CCBAU 051107T (KF962707, KF962683, JX437675)
0.10

## Slide 6
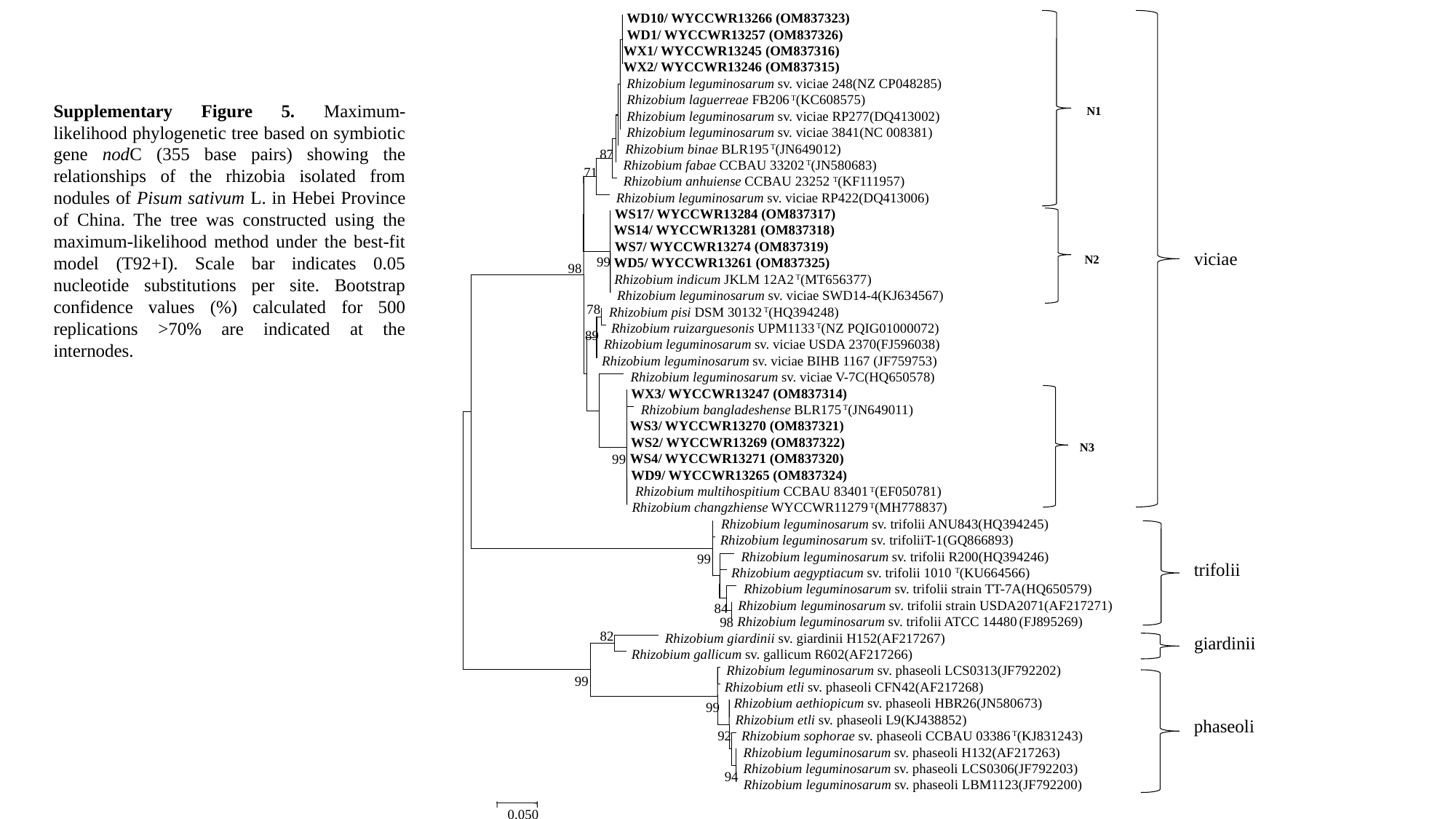

WD10/ WYCCWR13266 (OM837323)
 WD1/ WYCCWR13257 (OM837326)
WX1/ WYCCWR13245 (OM837316)
WX2/ WYCCWR13246 (OM837315)
 Rhizobium leguminosarum sv. viciae 248(NZ CP048285)
 Rhizobium laguerreae FB206 T(KC608575)
Supplementary Figure 5. Maximum-likelihood phylogenetic tree based on symbiotic gene nodC (355 base pairs) showing the relationships of the rhizobia isolated from nodules of Pisum sativum L. in Hebei Province of China. The tree was constructed using the maximum-likelihood method under the best-fit model (T92+I). Scale bar indicates 0.05 nucleotide substitutions per site. Bootstrap confidence values (%) calculated for 500 replications >70% are indicated at the internodes.
N1
 Rhizobium leguminosarum sv. viciae RP277(DQ413002)
 Rhizobium leguminosarum sv. viciae 3841(NC 008381)
 Rhizobium binae BLR195 T(JN649012)
87
 Rhizobium fabae CCBAU 33202 T(JN580683)
71
 Rhizobium anhuiense CCBAU 23252 T(KF111957)
 Rhizobium leguminosarum sv. viciae RP422(DQ413006)
 WS17/ WYCCWR13284 (OM837317)
WS14/ WYCCWR13281 (OM837318)
 WS7/ WYCCWR13274 (OM837319)
viciae
N2
99
WD5/ WYCCWR13261 (OM837325)
98
 Rhizobium indicum JKLM 12A2 T(MT656377)
 Rhizobium leguminosarum sv. viciae SWD14-4(KJ634567)
78
 Rhizobium pisi DSM 30132 T(HQ394248)
 Rhizobium ruizarguesonis UPM1133 T(NZ PQIG01000072)
89
 Rhizobium leguminosarum sv. viciae USDA 2370(FJ596038)
 Rhizobium leguminosarum sv. viciae BIHB 1167 (JF759753)
 Rhizobium leguminosarum sv. viciae V-7C(HQ650578)
 WX3/ WYCCWR13247 (OM837314)
 Rhizobium bangladeshense BLR175 T(JN649011)
WS3/ WYCCWR13270 (OM837321)
 WS2/ WYCCWR13269 (OM837322)
N3
WS4/ WYCCWR13271 (OM837320)
99
 WD9/ WYCCWR13265 (OM837324)
 Rhizobium multihospitium CCBAU 83401 T(EF050781)
 Rhizobium changzhiense WYCCWR11279 T(MH778837)
 Rhizobium leguminosarum sv. trifolii ANU843(HQ394245)
 Rhizobium leguminosarum sv. trifoliiT-1(GQ866893)
 Rhizobium leguminosarum sv. trifolii R200(HQ394246)
99
trifolii
 Rhizobium aegyptiacum sv. trifolii 1010 T(KU664566)
 Rhizobium leguminosarum sv. trifolii strain TT-7A(HQ650579)
 Rhizobium leguminosarum sv. trifolii strain USDA2071(AF217271)
84
 Rhizobium leguminosarum sv. trifolii ATCC 14480 (FJ895269)
98
giardinii
82
 Rhizobium giardinii sv. giardinii H152(AF217267)
 Rhizobium gallicum sv. gallicum R602(AF217266)
 Rhizobium leguminosarum sv. phaseoli LCS0313(JF792202)
99
 Rhizobium etli sv. phaseoli CFN42(AF217268)
 Rhizobium aethiopicum sv. phaseoli HBR26(JN580673)
99
phaseoli
 Rhizobium etli sv. phaseoli L9(KJ438852)
 Rhizobium sophorae sv. phaseoli CCBAU 03386 T(KJ831243)
92
 Rhizobium leguminosarum sv. phaseoli H132(AF217263)
 Rhizobium leguminosarum sv. phaseoli LCS0306(JF792203)
94
 Rhizobium leguminosarum sv. phaseoli LBM1123(JF792200)
0.050

## Slide 7
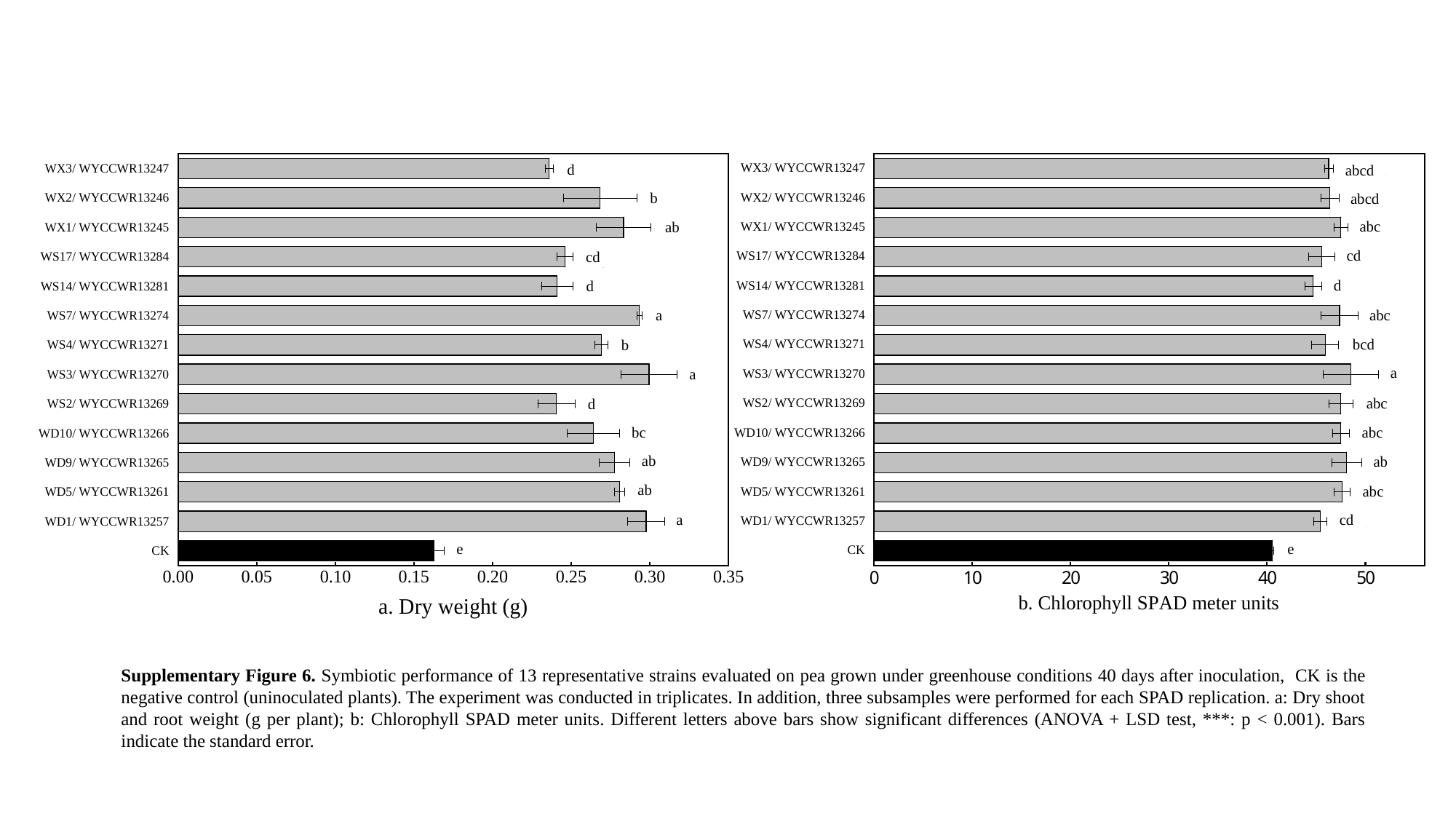

d
abcd
b
abcd
abc
ab
cd
cd
d
d
a
abc
bcd
b
a
a
abc
d
bc
abc
ab
ab
ab
abc
cd
a
e
e
Supplementary Figure 6. Symbiotic performance of 13 representative strains evaluated on pea grown under greenhouse conditions 40 days after inoculation, CK is the negative control (uninoculated plants). The experiment was conducted in triplicates. In addition, three subsamples were performed for each SPAD replication. a: Dry shoot and root weight (g per plant); b: Chlorophyll SPAD meter units. Different letters above bars show significant differences (ANOVA + LSD test, ***: p < 0.001). Bars indicate the standard error.
